# Injury Characteristics and Treatment Analysis of 166 Hospitalized Casualties in the Jishishan Earthquake

**DOI:** 10.1017/S1049023X2510157X

**Published:** 2025-12

**Authors:** Xuequan Wei, Mingyan Ma, Zhanlin Zhang, Xinting Lu, Xiaozhong Li, Yongdong An

**Affiliations:** 1. Nephrology Department, People’s Hospital of Linxia Hui Autonomous Prefecture, Linxia Hui Autonomous Prefecture, Gansu Province, China; 2. Public Health Management Department, People’s Hospital of Linxia Hui Autonomous Prefecture, Linxia Hui Autonomous Prefecture, Gansu Province, China; 3. Endocrinology Department, People’s Hospital of Linxia Hui Autonomous Prefecture, Linxia Hui Autonomous Prefecture, Gansu Province, China

**Keywords:** high-altitude, injury characteristics, Jishishan earthquake, medical treatment

## Abstract

**Introduction:**

In Jishishan County, Linxia Prefecture, Gansu Province, China, the altitude ranges from 1,787 meters to 4,308 meters. At 23:59 Beijing time on December 18, 2023, a magnitude 6.2 earthquake struck Jishishan County. The objective is to report the injury characteristics and medical treatments of those injured in the earthquake.

**Methods:**

The injury and treatment data were retrospectively collected and analyzed for earthquake-related injuries among patients admitted to the People’s Hospital of Linxia Hui Autonomous Prefecture and the Traditional Chinese Medicine Hospital of Linxia Hui Autonomous Prefecture.

**Observations:**

A total of 166 patients were hospitalized: 142 at the People’s Hospital of Linxia Hui Autonomous Prefecture and 24 at the Traditional Chinese Medicine Hospital of Linxia Hui Autonomous Prefecture. Among the injured, 40.3% presented with a single injury. The others had multiple injuries: 28.3% had two injuries, 14.5% had three injuries, 12.1% had four injuries, 4.2% presented with five injuries, while only 0.6% were diagnosed with six injuries. Additionally, 78.9% involved fractures alone, 36.8% involved lung contusions, and 34.9% involved both fractures and lung contusions. Conservative treatment was used slightly more than surgery (54.8% versus 45.2%). Among the 75 surgical cases, internal fixation and sutures were the most common (17.4% each). In total, 53.0% of the injured were treated and discharged and 47.0% were transferred to provincial hospitals. In addition, the outcome of injured patients with differing injury conditions was different.

**Analysis:**

Fractures and multiple injuries were the primary injury types in this study. Suturing and internal fixation were the most common surgical interventions. The core findings of this study provide an important reference for regionalized prevention and treatment of rural earthquake injuries in high-altitude regions.

## Introduction

An earthquake is a natural phenomenon characterized by the generation of seismic waves resulting from the rapid release of energy within the Earth’s crust.^1^ Statistical data indicate that over five million earthquakes occur globally each year. Among these, approximately 18 major earthquakes, classified as having a magnitude of 7.0 or higher, result in severe damage, with approximately one to two mega-earthquakes of a magnitude 8.0 or above each year.^2^ Earthquakes can result in extensive damage to buildings and lead to numerous casualties. Although earthquakes are a recurring phenomenon world-wide, emergency response efforts vary significantly across countries and regions, largely influenced by available resources and the feasibility of rescue and recovery operations.^3^

Jishishan County, located in Linxia Prefecture, Gansu Province, China has an altitude ranging from 1,787 meters to 4,308 meters.^4^ It is situated in the southeastern Ganshan region on the northeastern edge of the Tibetan Plateau, which is part of the north-south seismic zone.^5^ At 23:59 Beijing time on December 18, 2023, a magnitude 6.2 earthquake struck Jishishan County, with an epicenter depth of 10km.^6^ The epicenter of the earthquake was located within the secondary landmass in the southeastern part of the Qilian secondary landmass, where the fractures were along the northern and southern edges of the Laji Mountains converge, approximately 40km southwest of Linxia Terrace along the Inverted River-Linxia Fracture.^7^ Moreover, the high number of casualties and economic losses resulting from the earthquake can be attributed to the lack of seismic defenses in rural housing structures within the villages and towns at the epicenter, as well as the varied building ages that utilize different seismic codes.^8^

According to government reports, as of 13:00 on December 19, the earthquake resulted in 113 local fatalities and 536 injuries.^9^ Despite the severity of the event, there has been a notable lack of research on the types of injuries and treatments of earthquake casualties in Jishishan County. Consequently, the purpose of this study was to investigate the injury types and medical treatments of 166 earthquake casualties admitted at the People’s Hospital of Linxia Hui Autonomous Prefecture and the Traditional Chinese Medicine Hospital of Linxia Hui Autonomous Prefecture.

## Source

**Figure 1.**
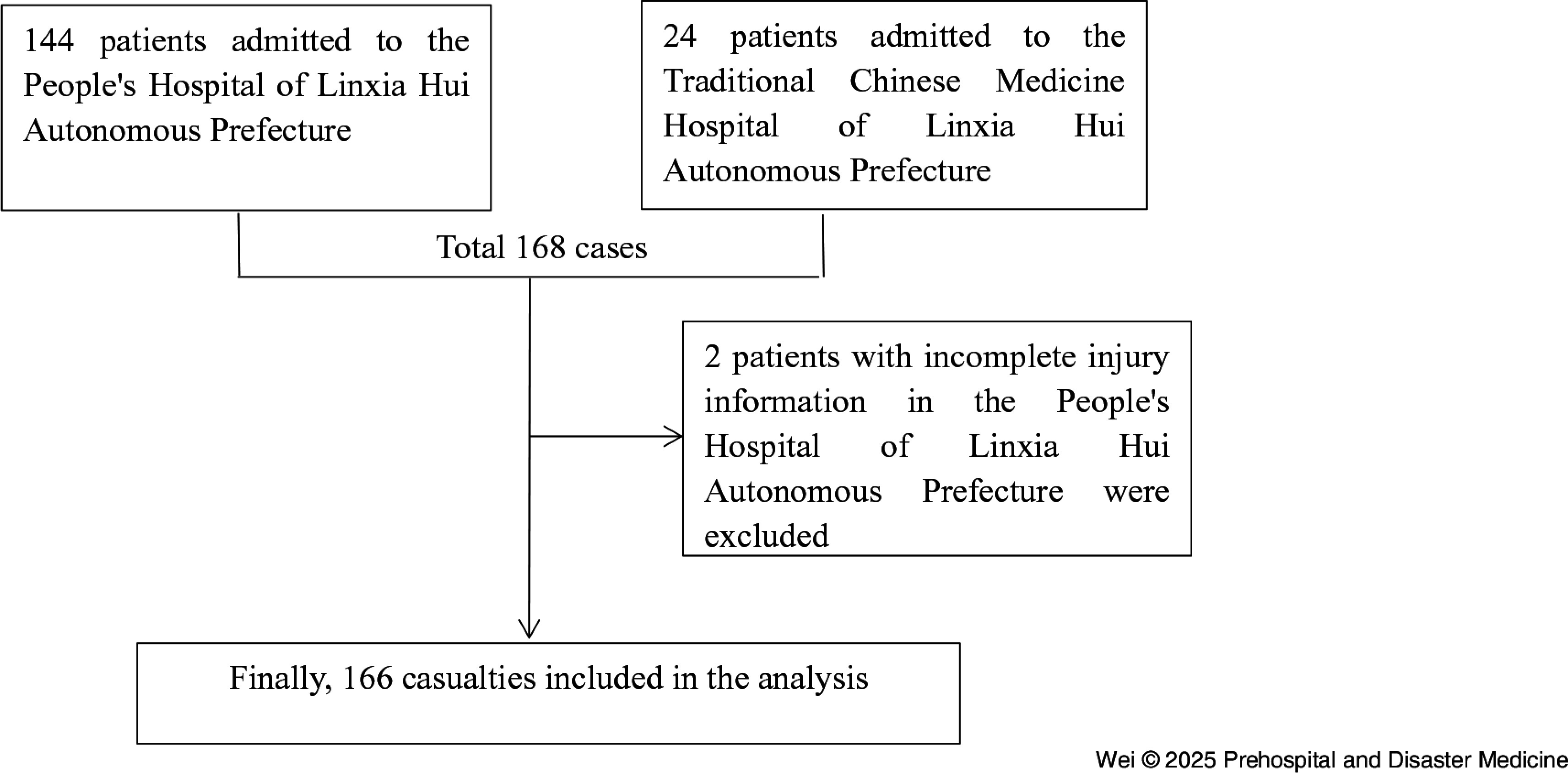
Data Inclusion/Exclusion Flow Diagram.

This study received approval from the Ethics Committee of the People’s Hospital of Linxia Hui Autonomous Prefecture (LZYY-LLSP-2024-01). This research began in March 2024. This study conducted a retrospective analysis of the clinical records of 166 patients with earthquake-related injuries admitted to the People’s Hospital of Linxia Hui Autonomous Prefecture and the Traditional Chinese Medicine Hospital of Linxia Hui Autonomous Prefecture following the 6.2 magnitude earthquake on December 18, 2023 in Jishishan (Figure 1).

### Research and Statistical Methods

This study is descriptive in nature and retrospectively collected information on the basic characteristics, injury types, treatment measures, surgical interventions, and prognosis among the 166 hospitalized earthquake casualties. The data analysis is mainly statistical description, and counting data are expressed by composition ratio.

### Study Site

The casualties from the earthquake were primarily admitted to the Grade 3 hospitals nearest to the epicenter: the People’s Hospital of Linxia Hui Autonomous Prefecture and the Traditional Chinese Medicine Hospital of Linxia Hui Autonomous Prefecture. These two hospitals played a crucial role in treating and transferring injured individuals following the Jishishan earthquake, and both have complete data on patient injuries and treatments.

### Study Variables

The injury category variable was derived from the discharge diagnosis recorded on the first page of the patients’ cases. The injury diagnoses were assigned according to the International Classification of Diseases (ICD)-10. The study used the discharge ICD-10 code and injury diagnosis in the analysis. The treatment measures variable was extracted from the treatment cost records within the Home Page of Medical Records. The surgical treatment program variables were derived from the names of surgeries documented in the Home Page of Medical Records. The outcome variables are classified as treated discharge and transfer to provincial hospital for treatment.

### Inclusion and Exclusion Criteria

Inclusion criteria included: (1) Jishishan earthquake casualties; (2) hospitalization; and (3) injury information was complete. Exclusion criteria included: injury information was incomplete.

### Definition

The following definitions were used in this study:

1. Green Channel: A fast-track service channel designed for specific groups or specific matters.
2. Principle of “Four Concentrations”: This refers to the centralized admission of patients, the centralized deployment of specialists, the centralized allocation of resources, and the centralized provision of treatment.

## Observations

### Basic Characteristics of Earthquake Casualties

This study included a total of 166 hospitalized casualties, comprising 142 patients admitted to the People’s Hospital of Linxia Hui Autonomous Prefecture and 24 patients at the Traditional Chinese Medicine Hospital of Linxia Hui Autonomous Prefecture. Among the injured, 54.2% were male, 78.3% were married, and 74.7% were farmers. Ages ranged from four months to 85 years, with 18 ∼ 65 years being the majority, accounting for 61.5%. The injured came from various ethnic groups, with Hui (44.1%) being the majority. This is detailed in Table 1.

**Table 1.** Distribution of Basic Characteristics for Earthquake Casualties

Variables		N (%)
Sex	Male	90 (54.2%)
	Female	76 (45.8%)
Age	<18	28 (16.9%)
	18∼65	102 (61.5%)
	>65	36 (21.6%)
Marital Status	Single	30 (18.1%)
	Married	130 (78.3%)
	Divorced	3 (1.8%)
	Widowed	3 (1.8%)
Nationality	Han	53 (31.9%)
	Hui	73 (44.1%)
	Dongxiang	14 (8.4%)
	Baoan	11 (6.6%)
	Salar	11 (6.6%)
	Tu	2 (1.2%)
	Tibetan	1 (0.6%)
	Tujia	1 (0.6%)
Occupation	Farmer	124 (74.7%)
	Student	16 (9.6%)
	Self-Employed	2 (1.2%)
	Freelance	2 (1.2%)
	Professional	3 (1.8%)
	Retiree	2 (1.2%)
	Others	17 (10.3%)

### Distribution of Injuries Category for Earthquake Casualties

Among the injured, 40.3% presented with a single injury. The most frequent injury was fractures (64.2%), followed by soft-tissue injuries (8.9%), neurological injuries (5.9%), preterm labor (3.0%), cerebral hemorrhage (3.0%), subcutaneous hematomas (3.0%), extrusion injuries (3.0%), visceral injuries (3.0%), skin injuries (3.0%), oculo-facial injury (1.5%), and pulmonary contusion (1.5%); Table 2.

**Table 2.** Distribution of Injuries Category for Earthquake Casualties

Injury Category	N (%)
Single Injury	67 (40.3%)
Pre-Term Labor	2 (3.0%)
Oculo-Facial Injuries	1 (1.5%)
Cerebral Hemorrhage	2 (3.0%)
Subcutaneous Hematomas	2 (3.0%)
Neurological Injuries	4 (5.9%)
Extrusion Injuries	2 (3.0%)
Visceral Injuries	2 (3.0%)
Skin Injuries	2 (3.0%)
Soft-Tissue Injuries	6 (8.9%)
Fracture	43 (64.2%)
Pulmonary Contusion	1 (1.5%)
Two Injuries	47 (28.3%)
Three Injuries	24 (14.5%)
Four Injuries	20 (12.1%)
Five Injuries	7 (4.2%)
Six Injuries	1 (0.6%)

In this study, 28.3% presented with two injuries; the most common combination was pulmonary contusion and fracture (44.7%). In this study, 14.5% presented with three injuries, and the most prevalent combination was pulmonary contusion, fracture, and soft tissue injury (25.0%). In this study, 12.1% presented with four injuries, and the most frequently encountered combination was pulmonary contusion, fracture, skin injury, and subcutaneous hematoma (20.0%). In this study, 4.2% presented with five injuries, most common among these was pulmonary contusion, fracture, nerve injury, subcutaneous hematoma, and cerebral hemorrhage which accounted for 28.6%. Lastly, only one (0.6%) was diagnosed with six injuries: pulmonary contusion, fractures, soft tissue injuries, skin injuries, crush injuries, and cerebral hemorrhage. Moreover, 78.9% presented with fractures and 36.8% with pulmonary contusions, while and 34.9% presented with a combination of fractures and pulmonary contusions in the total cases.

### Distribution of Treatment Measures for Earthquake Casualties

In the analysis of treatment approaches, 28.9% of earthquake victims received only Western medicine. Furthermore, 25.9% were treated with a combination of Chinese and Western medicine. Surgical interventions combined with Western medicine were administered to 22.9%, and 22.3% received surgical treatment combined with Chinese and Western medicines (Table 3).

**Table 3.** Distribution of Treatment Measures for Earthquake Casualties

Treatment Measures	N (%)
Western Medicine Treatment	48 (28.9%)
Chinese and Western Medicine Treatment	43 (25.9%)
Surgical and Western Medicine Treatment	38 (22.9%)
Surgical and Chinese and Western Medicine Treatment	37 (22.3%)

### Distribution of Surgical Treatment Items for Earthquake Casualties

In total, 75 cases (45.2%) of the earthquake victims required surgical treatment. Of these, 17.4% underwent internal fixation for fractures, with the surgical sites primarily involving three cases of ankle, three lumbar vertebrae, two patellar, one femoral, one radius, one rib, one metatarsal, and one case involving both the patella and phalanges. Additionally, sutures accounted for 17.4%, with the surgical sites primarily encompassing nine cases of skin, three scalp, and one forearm tendon. There were 12.0% that required arterial puncture, alongside 6.7% of traction treatment, resections, central venous cannulation, and arterial puncture. This is detailed in Table 4.

**Table 4.** Distribution of Surgical Treatment Items for Earthquake Casualties

Surgical Treatment Items	N (%)
Bladder Repair	2 (2.7%)
Traction Treatment	5 (6.7%)
Plaster Bandage Fixation	2 (2.7%)
Internal Fixation of Fracture	13 (17.4%)
Manipulative Reduction of Fracture of the Lower End of the Radius	2 (2.7%)
Resection Treatment	5 (6.7%)
Debridement	2 (2.7%)
Excision and Debridement of Skin and Subcutaneous Necrotic Tissue and Ulnar Nerve Exploration	1 (1.3%)
Arterial Puncture	9 (12.0%)
Central Venous Cannulation and Arterial Puncture	5 (6.7%)
Central Venous Cannulation by Internal Jugular Vein Puncture	3 (4.0%)
Suture	13 (17.4%)
Forearm Muscle Suture and Ulnar Artery Anastomosis	1 (1.3%)
Gastric Intubation Decompression	1 (1.3%)
Closed Drainage of the Chest Cavity	2 (2.7%)
Percutaneous Balloon Dilatation of Vertebral Body	1 (1.3%)
Exploratory Laparotomy and Exploratory Bladder and Jugular Vein Puncture Central Venous Catheterization	1 (1.3%)
Perineal Laceration Repair and Cephalic Vaginal Midwifery and Partial Breech Traction	1 (1.3%)
Eyeball Rupture Repair and Eyelid Laceration Suture Surgery and Vitreous Drug Injection	1 (1.3%)
Intracerebral Hematoma Removal and Craniotomy Decompression and Ventilator Treatment and Arterial Puncture	1 (1.3%)
Closed Thoracic Drainage and Internal Jugular Vein Puncture Central Venous Catheterization and Arterial Puncture	1 (1.3%)
Exploratory Laparotomy and Hepatic Rupture Repair and Bile Duct Repair and Intestinal Adhesion Release and Arterial Puncture	1 (1.3%)
Lumbosacral Posterior Column Fusion, Posterior Approach and Pedicle Nail Fixation and Spinal Canal Decompression and Allograft Bone Grafting	1 (1.3%)
Internal Jugular Vein Puncture Central Vein Catheterization and Arterial Puncture and Femoral Vein Puncture Catheterization and Closed Thoracic Drainage	1 (1.3%)

### Distribution of Outcomes for Earthquake Casualties

In this study, 53.0% were treated and discharged from the hospital and 47.0% were transferred to provincial hospitals for on-going treatment (Table 5). In addition, the outcome of injured patients with differing injury conditions was different, as detailed in Table 6.

**Table 5.** Distribution of Outcomes for Earthquake Casualties

Outcomes	N (%)
Treated Discharge	88 (53.0%)
Transfer to Provincial Hospital for Treatment	78 (47.0%)

**Table 6.** Outcome Distribution of Different Injury Conditions

	Injuries					
	Single Injury	Two Injuries	Three Injuries	Four Injuries	Five Injuries	Six Injuries
	(n = 67)	(n = 47)	(n = 24)	(n = 20)	(n = 7)	(n = 1)
Outcome						
Treated Discharge	44 (65.7%)	23 (48.9%)	11 (45.8%)	10 (50.0%)	0 (0.0%)	0 (0.0%)
Transfer to Provincial Hospital for Treatment	23 (34.3%)	24 (51.1%)	13 (54.2%)	10 (50.0%)	7 (100.0%)	1 (100.0%)

## Analysis

In this study, the data revealed a slightly higher prevalence of males compared to females. Additionally, a significant proportion of the injured fell within the 18-65 age group (61.5%), which may be associated with varying levels of exposure and behavior during the earthquake event. There are Han, Baoan, Dongxiang, Salar, Hui, Tu, and Tibetan ethnic groups in Jishishan County.^4^ The injured individuals in this study included several ethnic groups, with the Hui group making up the largest proportion at 44.1%. Furthermore, farmers made up the majority of the injured population, likely because the earthquake’s epicenter was primarily located in rural areas, where buildings are generally less resilient to seismic activity.

The injury categories among earthquake casualties in this study were multifaceted. A systematic review indicated that multiple injuries are a common feature among earthquake casualties, with approximately one-third to one-half of these individuals presenting with more than one type of injury.^10^ Zhao’s retrospective study of the Wenchuan earthquake (2008) highlighted that multiple injuries were a significant characteristic, with a maximum of five combined injuries recorded.^11^ Kang, et al’s investigation on the Lushan earthquake (2013) found that the most common multiple injuries were soft tissue contusions and lacerations, and only 11.2% of the wounded had multiple injuries of Type 3 or above.^12^ In the study, two injuries accounted for 28.3%, three injuries represented 14.5%, four injuries accounted for 12.1%, 4.2% had five injuries, and only 0.6% had six injuries. This indicated that multiple injuries are a critical feature among those injured in this earthquake, with the highest observed combination being six injuries.

The analysis revealed that 78.9% of earthquake casualties involved fractures, confirming that fractures were the predominant type of injury in this case. This finding aligns with reports indicating a higher incidence of fractures in studies of other major earthquakes, such as those in Wenchuan, Yushu (2010), Lushan, and Nepal (2015).^11–14^ One study identified pulmonary contusion as a reliable indicator of chest injury severity,^15^ while another report noted that chest crush injuries sustained during the Sichuan earthquake (2008) were life-threatening and could lead to multiple fractures and lung injuries.^16^ In the research, 36.8% of earthquake casualties had pulmonary contusion, and 34.9% presented with both fractures and pulmonary contusion. Research indicates that the incidence of fractures and related injuries in earthquakes is associated with patients’ body postures and living conditions at the time of the earthquake.^17^ Since this earthquake occurred at 23:59 on a cold winter night, many individuals were asleep. This may have led to reduced earthquake perception and emergency response capabilities, exacerbated by a subsequent power outage.

In this study, 45.2% of participants underwent surgical treatment, which primarily involved internal fixation of fractures and suturing. Studies of prior earthquakes, including the Wenchuan and Lushan earthquakes, have identified internal fixation and suturing as the most common surgical interventions.^12,18^ Furthermore, surgical treatment of fractures has been recognized as a primary treatment strategy in various earthquake scenarios, such as the Bam earthquake (2003), the Haiti earthquake (2010), the Nepal earthquake, and the Jiuzhaigou earthquake (2017) in Sichuan Province.^15,19–21^

Experience in providing medical treatment to earthquakes victims shows that the critical period for effective care is within the first 72 hours after the disaster.^22^ The People’s Hospital of Linxia Hui Autonomous Prefecture and the Traditional Chinese Medicine Hospital of Linxia Hui Autonomous Prefecture sent a medical rescue team to the earthquake-hit area immediately after the earthquake to carry out on-site treatment, screening, transfer of the injured, and psychological counseling. They were the first to admit and treat the injured transferred from Jishishan County. The emergency department opened a green channel, conducted pre-screening, and triage. Following the principle of “Four Concentrations,” a quasi-intensive-care-unit ward was rapidly established. Moving forward, it is crucial to focus on advancing and enhancing medical technologies and capabilities, enabling more effective responses to future public health emergencies.

To prevent earthquakes in high-altitude cold areas, a comprehensive “anti-seismic and cold-resistant” strategy is necessary. This involves combining local building materials, traditional craftsmanship, and modern technology to enhance building resilience and seismic performance, while also optimizing the emergency response system. Medical treatment should be based on the principle of laddering: first-level response (the golden 72 hours), with priority given to patients experiencing hypoxia combined with traumatic shock. Secondary triage, along with multidisciplinary collaboration, is essential for treating the injured. In addition, post-disaster psychological intervention is necessary.

The primary characteristics of injuries sustained by earthquake casualties in this study included fractures and multiple traumas. Suturing and internal fixation were the most common surgical interventions. The core findings of this study provide an important reference for regionalized prevention and treatment of earthquake injury in high-altitude regions. This will enhance the response capabilities of regional hospitals in handing earthquake disasters.

The limitations of this study include: (1) the sample of the injured only covers the cases admitted to tertiary hospitals, which may introduce selection bias; (2) currently, there is no comparable data on earthquake casualties available for analysis; and (3) due to limited information on earthquake victims and the complexity of post-earthquake reconstruction efforts, follow-up work for the injured is difficult to carry out, leading to a lack of follow-up data.
